# Harnessing the ubiquitin code to respond to environmental cues

**DOI:** 10.1042/EBC20210094

**Published:** 2022-08-05

**Authors:** Beatriz Orosa-Puente, Steven H. Spoel

**Affiliations:** Institute of Molecular Plant Sciences, School of Biological Sciences, University of Edinburgh, Edinburgh EH16 5JF, U.K.

**Keywords:** chain topology, E2 enzyme, E3 ligase, plant stress responses, proteasome, ubiquitin

## Abstract

Ubiquitination is an essential post-translational signal that allows cells to adapt and respond to environmental stimuli. Substrate modifications range from a single ubiquitin molecule to complex polyubiquitin chains, where diverse chain topologies constitute a code that is utilized to modify the functions of proteins in numerous cellular signalling pathways. Diverse ubiquitin chain topologies are generated by linking the C-terminus of ubiquitin to one of seven lysine residues or the N-terminal methionine 1 residue of the preceding ubiquitin. Cooperative action between a large array of E2 conjugating and E3 ligase enzymes supports the formation of not only homotypic ubiquitin chains but also heterotypic mixed or branched chains. This complex array of chain topologies is recognized by proteins containing linkage-specific ubiquitin-binding domains and regulates numerous cellular pathways. Although many functions of the ubiquitin code in plants remain unknown, recent work suggests that specific chain topologies are associated with particular molecular processes. Deciphering the ubiquitin code and how plants utilize it to cope with the changing environment is essential to understand the regulatory mechanisms that underpin myriad stress responses and establishment of environmental tolerance.

## Introduction

**Figure 1:**
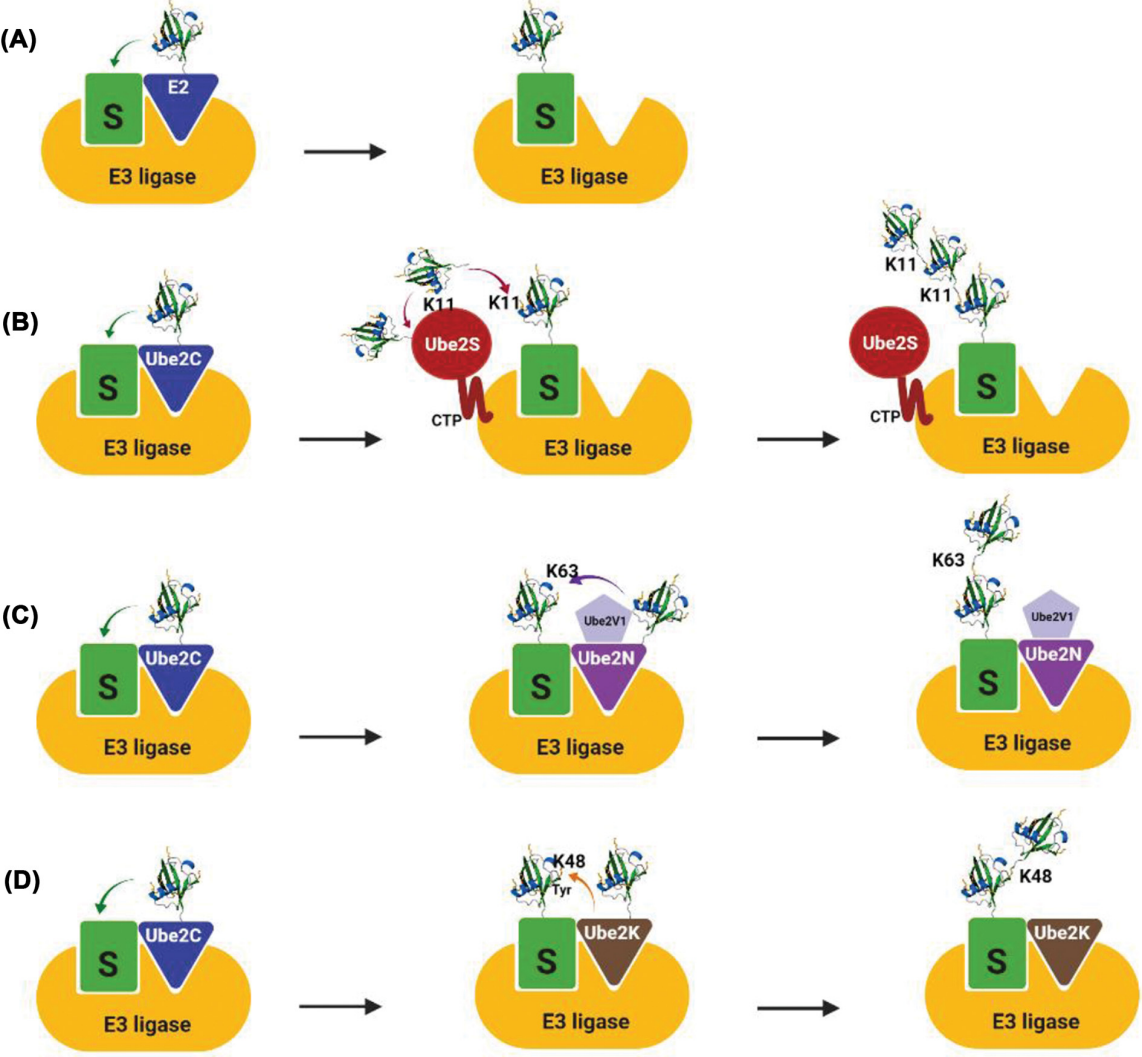
The mechanistic basis of linkage-specific ubiquitin chain formation by E2 and E3 enzymes Polyubiquitination of substrates involves multiple steps and can involve multiple E2 enzymes that mono- or polyubiquitinate substrates. (**A**) Some E2–E3 combinations can only monoubiquitinate the substrate (S). (**B**) Linkage-specific ubiquitin chains are generated by several specialized mechanisms. After monoubiquitination of the substrate by Ube2C, the E2 enzyme Ube2S is recruited and associates with both a donor and acceptor ubiquitin. Whereas the donor ubiquitin is bound via both a thioester and non-covalent interactions, the acceptor ubiquitin is bound near its K11 residue through electrostatic interactions. Ube2S then facilitates a reaction in which K11 from the acceptor ubiquitin attacks the thioester bond between Ube2S and the donor ubiquitin. This results in formation of K11-linked di-ubiquitin that is then utilized to elongate ubiquitin chains of the substrate. (**C**) By contrast, Ube2N utilizes the tightly bound E2-like subunit Ube2V1 (a pseudo E2 that lacks the conserved cysteine residue critical for the catalytic activity of E2 enzymes) to favourably position the K63 side chain of the incoming ubiquitin, whereas Ube2K interacts with a tyrosine residue near K48 of the acceptor ubiquitin to promote K48-linked chain formation (**D**).

Ubiquitination is the process of conjugation of ubiquitin to substrate proteins, most often through an iso-peptide bond between the C- terminus of ubiquitin and an ε-amino group of a lysine residue of the substrate. Ubiquitin can also be conjugated to other amino acids, such as cysteine, serine or threonine [1,2] or to the N-terminal methionine [3]. Ubiquitin sequence and structure are highly conserved between animals, plants and fungi, suggesting that ubiquitin from different species may be functionally interchangeable [4]. Ubiquitination is essential for proper cell function, regulating major processes from metabolism and signal transduction to stress responses across kingdoms. The essential nature of this protein modification is connected to its versatility: ubiquitin can occur as a monomeric modification or can generate complex chains through ligation of ubiquitin to one or more residues of the preceding ubiquitin This process can create numerous chain topologies (i.e*.* homotypic, heterotypic, branched), known as the ‘ubiquitin code’, which each have different functional consequences (Figure 1A). Homotypic chains are characterized by a single predominant linkage, while heterotypic ubiquitin chains contain multiple linkage types and adopt mixed or branched topologies. In a mixed chain, a number of different ubiquitin linkages are connected to one another without branching the chain, while in branched chains some ubiquitin molecules are modified on two or more residues with additional ubiquitins [5]. The ubiquitin code is ‘written’ by a combination of E1 ubiquitin-activating enzymes, E2 ubiquitin-conjugating enzymes and E3 ubiquitin ligases, ‘erased’ by various deubiquitinases (DUBs) and ‘decoded’ by proteins harboring ubiquitin-binding domains (UBDs) [5]. Each of these players is essential for a specific polyubiquitin chain to act as a distinct intracellular signal. In Arabidopsis, well over a thousand genes encode for members of the ubiquitin machinery, representing one of the most elaborate and prevalent regulatory mechanisms in plants. Curiously, the number of genes that code for each enzyme family is highly variable, suggesting that certain enzymes have higher specificity and greater versatility than others. The Arabidopsis genome harbors two E1 enzymes, 37 E2s, over 1500 proteins that function as single subunit E3 ubiquitin ligases or as components of E3 complexes, and 56 DUBs. This suggests that E3 ligases are primarily responsible for much of the specificity and versatility of the ubiquitin code in plants.

Ubiquitination is initiated by an ATP-dependent E1 enzyme, which captures ubiquitin via its active site cysteine residue, forming a thioester bond with the C-terminus of ubiquitin [6]. Ubiquitin is then transferred to a cysteine residue of an E2 enzyme [7–9], which together with E3 ligases ubiquitinate a specific substrate [8,9]. As a result, an isopeptide linkage is formed between the C-terminal carboxyl group of a ubiquitin moiety and an ε-NH_2_ group of a lysine of the substrate. Hence, E2–E3 complexes can ubiquitinate substrates with either a single ubiquitin moiety (mono-), several single ubiquitin moieties (multimono-), or chains of ubiquitin (polyubiquitination) via addition of ubiquitin to one of seven lysine residues (K6, K11, K27, K29, K33, K48 and K63) or the N-terminal methionine (M1) of a preceding ubiquitin moiety already attached to the target substrate.

## How do E2–E3 complexes assemble diverse ubiquitin chain topologies?

Each of the 37 E2 enzymes encoded by the Arabidopsis genome is thought to be responsible for a different type of ubiquitin topology and interacts with different E3 enzymes that provide substrate specificity [4]. Therefore, E2 enzymes can be characterized in terms of their molecular activities: (i) E2s that transfer a single ubiquitin on to a target protein residue (monoubiquitylating E2s) (Figure 1A), (ii) E2s that transfer ubiquitin onto another ubiquitin (chain-building E2s) and (iii) non-selective E2s that can do either [8]. E2 enzymes that catalyse the formation of specific ubiquitin linkage topologies have evolved to recognize and position particular lysine residues within the structural and/or sequence context of ubiquitin. For example, mammalian Ube2S interacts with both a donor and acceptor ubiquitin. While the donor ubiquitin is bound via both a thioester and non-covalent interactions, the acceptor ubiquitin is bound near its K11 residue through electrostatic interactions. Ube2S then catalyses a reaction in which K11 from the acceptor ubiquitin attacks the thioester bond between Ube2S and the donor ubiquitin, resulting in formation of K11-linked di-ubiquitin that is subsequently transferred to elongate ubiquitin chains of the substrate (Figure 1B) [10,11]. Using a different mechanism, Ube2N heterodimerizes with the E2-like subunits, Ube2V1 or Ube2V2, to favourably position the K63 side chain of the incoming ubiquitin (Figure 1C) [12]. Similarly, Ube2K has a unique region near its active site that interacts with a tyrosine adjacent to K48 of the acceptor ubiquitin to catalyse K48-linked polyubiquitination (Figure 1D) [8].

E3 ligases are arguably the most important players in ubiquitin chain formation since they position both the E2 enzyme and the substrate in close proximity to each other. Considering that most proteins in the cell are thought to be subject to ubiquitination, this potentially immense substrate repertoire is underpinned by a high diversity of E3 ligases. Indeed, during eukaryotic evolution, E3 ligase families have expanded massively. They are generally divided into three mechanistic classes: RING (really interesting new gene)/U-box, HECT (homologous to E6AP C-terminal) and RBR (RING-between-C-terminal) ligases [8,13]. RING/U-box ligases are the largest of these classes with 508 single subunit RING-type and 64 U-box-type enzymes in Arabidopsis that directly catalyse the transfer of ubiquitin from the E2 enzyme to a substrate [14,15]. By contrast, the Arabidopsis genome encodes for only 7 HECT-type ([16,17], and reviewed in this issue by Wang and Spoel) and 40 RBR-type ligases [18–20]. These classes of ligases employ a two-step mechanism, in which ubiquitin is first transferred from an E2 enzyme to an active site cysteine of the E3, producing an thioester-linked E3-ubiquitin intermediate, before being transferred to a substrate [18–21].

So what role do E3 ligases play in generating the ubiquitin code? Monoubiquitination and polyubiquitination reactions catalysed by E3 enzymes present significant challenges. During the monoubiquitination step, ubiquitin binds directly to a substrate protein. For this process to be site-specific, correct positioning of substrate’s intended lysine(s) towards the E2/E3 active site is required [22–25]. In most cases, monoubiquitination can occur at multiple lysines with little dependence on sequence context [26,27]; indeed, mutation of a ubiquitinated lysine is often insufficient to prevent ubiquitination of the substrate if adjacent lysines are available. In contrast, ubiquitin chain elongation often occurs with specificity for the amino acid group of the acceptor ubiquitin [27,28]. Thus, to achieve linkage specificity during sequential addition of ubiquitins, an E3 ligase must repeatedly position the distal acceptor ubiquitin molecule on a growing chain with high precision. E3 enzymes have developed different mechanisms to meet this demand: (i) by using two different E2s for priming and chain extension [29,30], (ii) by recruiting E2s with different linkage-type specificities [8], (iii) by cooperating with another E3 ligase as found for the mammalian HECT-type E3 ligase, HUWE1, which adds K48-linked chains to K63-linked chains preassembled by TRAF6, transforming the ubiquitin-dependent signal from non-proteolytic to proteolytic [27,31,32], and (iv) by cooperating with E4 ligases that compared with E3 ligases, are more efficient in extending polyubiquitin chains, as reported for the plant immune coactivator NPR1 [33–36].

## Sequential versus en bloc ubiquitin chain assembly

**Figure 2:**
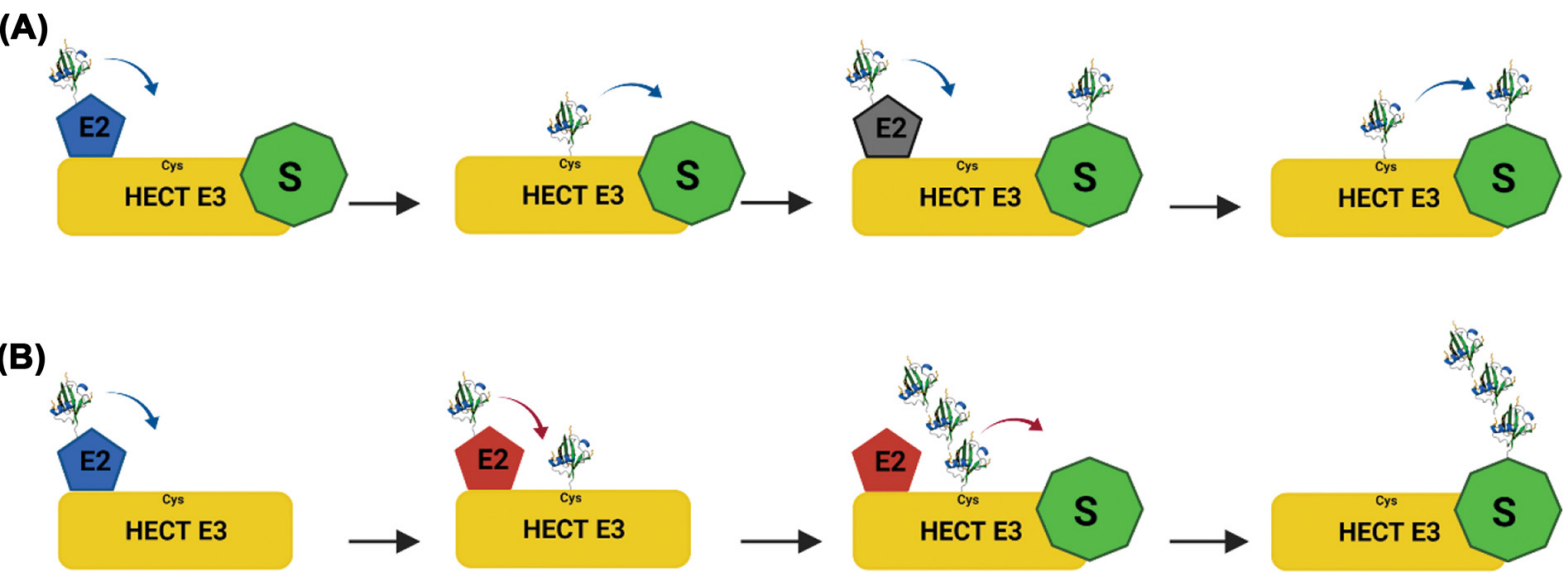
Sequential addition versus en bloc ubiquitination by HECT-type E3 ligases The E2 binds to the HECT-E3 domain and transfers ubiquitin to the active site cysteine via a thioester bond from which it is transferred to the substrate protein. HECT domains use different mechanisms of chain elongation either by transferring individual ubiquitin molecules to a growing substrate-linked chain (**A**) or by pre-assembling chains on its active site cysteine before transferring the whole chain to a substrate (**B**).

Ubiquitin can be added to substrates by ‘sequential addition’ or ‘en bloc transfer’, the mechanisms of which differ with respect to processivity of ubiquitin chain formation (Figure 2). Sequential addition is believed to be the predominant mechanism used by E2–E3 complexes and requires the step-wise transfer of individual ubiquitin molecules to the end of a chain. By contrast, the *en bloc* mechanism involves transferring entire chains that have been pre-formed on the active-site cysteine of an E2 or HECT/RBR E3 to a substrate (Figure 2) (reviewed in [27,37]).

Why are there two different mechanisms for assembly of ubiquitin chains? While kinetic analysis revealed that most encounters between E3 ligases and substrates are unproductive, once the substrate has been monoubiquitinated the rate of subsequent ubiquitin additions outcompetes substrate dissociation. However, lag time of ubiquitin transfer then begins to increase proportional to the length of the chain, thereby limiting chain growth [38]. Nonetheless, sequential transfer of ubiquitin allows some E3 ligases to construct heterotypic chains. For example, mammalian WWP1 catalyses the formation of non-proteolytic K63-linked chains until the length reaches ∼4 ubiquitins in length, before switching to build chains linked through K11 and K48, which promote proteasomal degradation of the modified substrate [39]. Even though sequential addition of ubiquitin provides versatility to rapidly change chain topology based on cellular inputs, en bloc transfer of entire pre-assembled ubiquitin chains may be a faster and more efficient mechanism for substrate modification. One of the best studied E3 ligases that utilizes an *en bloc* transfer mechanism is the HECT-type UBE3A (E6AP) ligase. UBE3A contains two distinct E2 docking sites. The first site positions the E2∼ubiquitin conjugate close to its active site cysteine residue, resulting in transfer of ubiquitin to generate a UBE3A∼ubiquitin thioester. The second site then engages further E2∼ubiquitin conjugates to build an active site-anchored K48-linked chain that is subsequently transferred en bloc to the substrate (Figure 2B) [40]. Similarly, some E2 enzymes can build a pre-formed ubiquitin chain on their own active sites cysteine, allowing them to generate unanchored ubiquitin chains, but it remains unclear if these can be transferred en bloc to substrates [41]. While en bloc transfer of ubiquitin chains has so far only been associated with formation of homotypic chains, it is conceivable that this mechanism could also generate long heterotypic or branched ubiquitin chains.

## Decoding the complexity of diverse ubiquitin chain topologies

**Figure 3:**
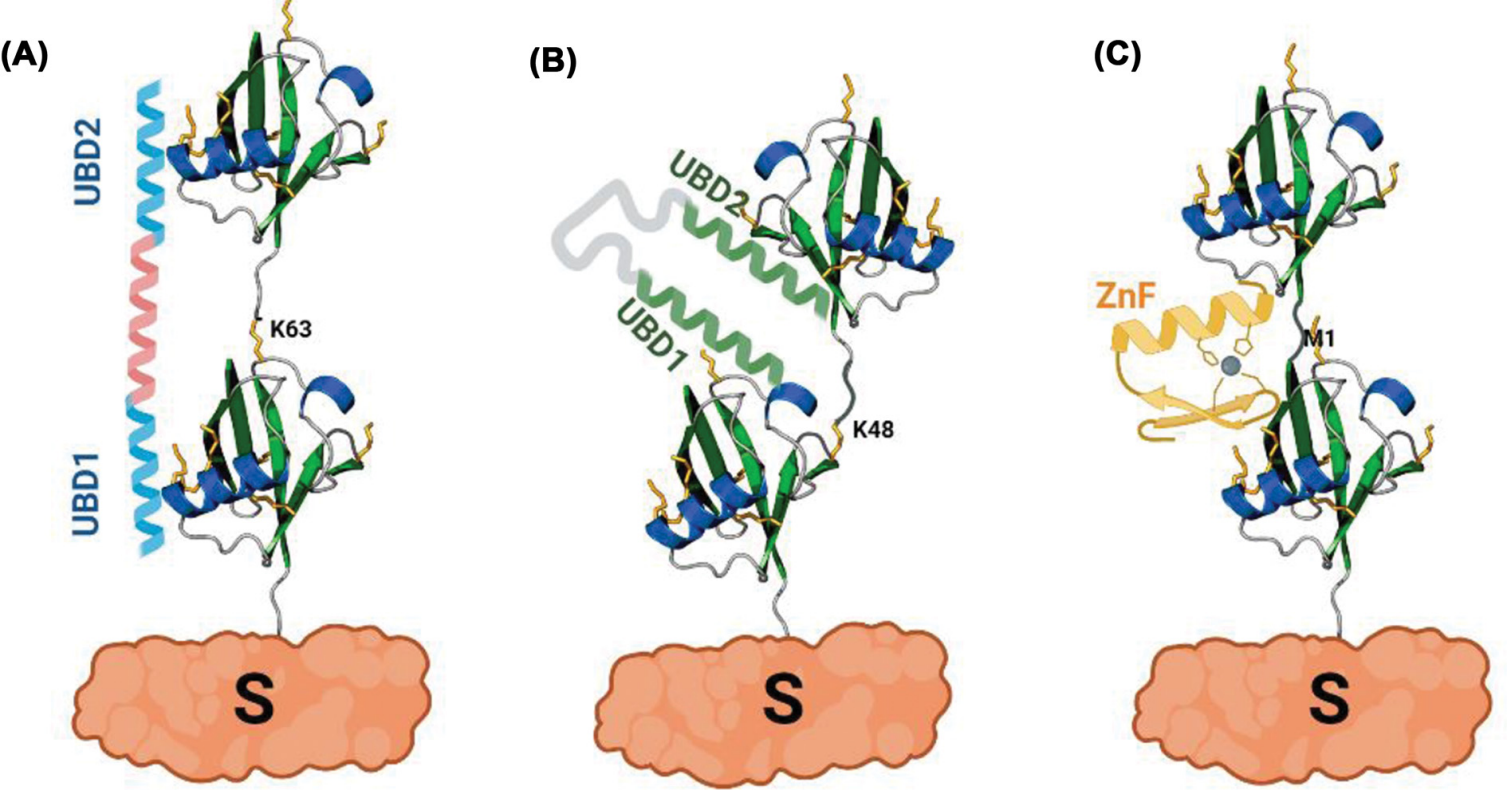
The ubiquitin code is recognized by proteins containing single or multiple ubiquitin-binding domains (UBDs) UBDs are structurally and functionally diverse, reflecting the diversity of the ubiquitin code. Based on their structures, UBDs differ in the cooperative binding of ubiquitin. For example, a combination of two UBDs can be used to specifically recognize K63- (**A**) or K48-linked (**B**) chains, while a single zinc finger-containing UBDs recognizes M1-linked di-ubiquitin (**C**).

Structural analyses of ubiquitin linkage types revealed that different chains adopt distinct conformations that can be classified as ‘open’ or ‘compact’. For example, the compact conformation adopted by K6-, K11- and K48-linked di-ubiquitin, in which an intermolecular interface is present between the distal and proximal ubiquitin (Figure 3B), contrasts with the extended conformation of M1- and K63-linked polyubiquitin where besides the linker, there is no contact between two consecutive ubiquitin moieties (Figure 3A,C) [42,43]. These contrasting conformations are recognized by proteins containing a single or multiple UBDs. UBD-containing proteins exhibit binding affinities specific to ubiquitin chain topology, conformation and length, and can decode or translate them into cellular signals. Consequently, different ubiquitin chain topologies are associated with distinct modulations of protein function. For example, they can control the stability, activity, protein–protein interactions or localization of substrates. Despite our efforts to understand the ubiquitin code, for some linkages only a few targets have been described so far, and therefore we still do not fully comprehend their role in cell regulation.

While the function of most linkages is not known in plants, studies in other eukaryotes can provide insight into their potential role in plants. Studies in mammalian cells show involvement of K6-linked chains in autophagy and DNA damage response. Moreover, upon viral infection, the transcription factor IRF3 (interferon regulatory factor 3) is modified with K6-linked conjugates, which stimulates it to induce immune gene expression. K11- and K48-linked chains are associated with proteasome-mediated degradation and either of these homotypic conjugates can initiate protein degradation on their own [44,45]. Nonetheless, their concurrent incorporation into substrates as separate or mixed/branched chains, strongly enhances recognition by proteasomal ubiquitin receptors. K27 linkages are a major player in innate immunity, especially through regulation of key viral immune response regulators. In this context, a member of a family of mammalian RING-containing TRIM (Tripartite motif) ligases assembles K27-linked chains that, through auto- or self-ubiquitination induce its GTP hydrolysis activity and signal for selective autophagy [46]. Even though the molecular function of K29 is unclear, the amount of K29-linked polyubiquitin increases following proteasome inhibition, and therefore it has been linked to the degradation pathway. Furthermore, ubiquitination with K29/K33‐linked mixed chains has been associated with regulation of protein kinases and innate immunity [43,44]. The molecular role of K33 linkage type is one of the least understood [44]. Nonetheless, K33-linked chains are likely non-proteolytic, because this chain topology does not significantly accumulate upon proteasome inhibition [43,44]. K48-linked chains are the most abundant linkage type and target the ubiquitinated substrate for proteasome-mediated degradation. Indeed, the proteasome contains ubiquitin receptors that preferentially recognize K48-linked topologies [47]. K63-linked chains are the second most abundant chain topology in cells. They are involved in endocytosis, innate immunity, and more recently, they have been shown to regulate DNA damage repair and transcriptional regulation [48]. In addition to Lys-linked polyubiquitin chains, ubiquitin can also be attached to the N-terminal Met1 (M1-linked), generating linear polyubiquitin chains. M1-linked chains are much less abundant than any other type of polyubiquitin chains, even though they have pivotal roles in cell survival, proliferation and the immune response [49,50]. M1 is specifically generated by the E3 ligase complex, linear ubiquitin chain assembly complex (LUBAC) [49,50]. So far, M1-linked ubiquitin chains and corresponding E3 ligase complexes have not been found in plants.

## Ubiquitin chain topologies in plant cell signalling

Although ubiquitin signalling has been studied extensively in context of plant cell signalling, little remains known about the specific roles of diverse ubiquitin chain topologies. Proteomic analysis of the Arabidopsis ubiquitome identified footprints for each of the seven Lysine-linked chains (K6, K11, K27, K29, K33, K48 and K63), where proteolytic K48 linkages were most abundant, comprising ∼30% of the total detected linkages [51,52]. In response to environmental stimuli, proteasome-mediated degradation acts as a central regulator of most phytohormone signalling pathways, including salicylic acid (SA), jasmonic acid (JA), gibberellic acid (GA), auxin, brassinosteroids (BR), abscisic acid (ABA) and ethylene [14]. In these pathways the proteasome regulates the stability of signalling proteins as well as many transcriptional activators and repressors. Indeed, proteasome-mediated degradation of repressors activates many hormone-responsive transcriptional programmes, whereas degradation of activators can both promote and limit target gene expression [35,53]. Even though limited proof is available, it is expected that most of these proteolytic events are regulated by K48-linked ubiquitin chains. In fact, after treating Arabidopsis seedlings with the proteasome inhibitor MG132, more than half of the ubiquitinated proteins increased their ubiquitination level, suggesting that K48-linked modifications have dramatic impacts on the plant proteome [51]. Ubiquitin chains linked through K63 residues are the second most abundant linkage type in plants [51,52]. Insights into K63-mediated signalling have been derived from characterization of Arabidopsis mutants of the K63-assembling E2 ligases, *UBC35*, *UBC36* and *UEV1D* [54–56], as well as by using a K63 polyubiquitin sensor based on yeast VPS27 that specifically recognizes K63-linked Ub chains [56]. This revealed that K63-linked chains play a role in a wide array of cellular and physiological functions as sustaining translation efficiency under stress, nuclear import, splicing and DNA structure/topology. Strikingly, many membrane proteins are also modified by K63 linkages, which often signals for their internalization and intracellular trafficking [56]. Furthermore, PARylation and K63-linked ubiquitination coordinately regulate pathogen pattern-triggered immunity [57]. Moreover, the interaction between Fen (tomato protein kinase Fen) and Fni3 (a Ubc13 ortholog) is necessary for Fen-triggered programmed cell death [58], strengthening the connection between K63 linkages and perception of plant immunity.

K6, K11, K27, K29 and K33 linkages are much less abundant in plant cells [51,52]. K29 has been implicated in targeting GA-responsive DELLA proteins for proteasomal degradation and in regulating various environmental responses, including to light, temperature and water [59]. Other atypical linkages such as K6, K11 and K33 have not been well characterized in plants. However, the reduction of K27 and K29 peptides in response to the bacterial peptide, flagellin 22, provides a clue that these linkage types either interfere with pattern-triggered immune signalling or are involved in developmental signalling pathways that are suppressed upon activation of immunity [52]. Moreover, K11-linked chains have been associated with female gametophyte development [60]. To date, M1-linked chains have not been identified in plants. Thus, further studies are needed to uncover the functions of the diverse ubiquitin chain topologies in regulating plant cell signalling and the E3 ligases that assemble them.

Additional complexity in ubiquitin signalling is achieved through the formation of heterotypic ubiquitin chains, which contain multiple ubiquitin linkage types and adopt mixed or branched topologies [61,62]. Heterotypic linkages can be formed by the sequential cooperation between two E3 ligases [32], or by shifting the linkage specificity of a unique E3 ligase [40]. By further remodelling of the ubiquitin chain, cells are able to redirect the functionality of the substrate to real-time environmental inputs. For example, K63/M1-linked hybrid chains activate mammalian innate immunity by colocalizing K63- and M1-associated immune complexes [63], while K63-K48 branched chains amplify the outputs of the NF-κB immune activator [32]. Moreover, K11/K63 linkages initiate more efficient endocytosis of some proteins [64]. However, K11, K29 and K63 non-degradative linkage types can also trigger proteasome-mediated degradation of substrates by serving as a ‘seed’ for K11/K48, K29/K48 and K63/K48 branched ubiquitin chains that are recognized by proteasomal ubiquitin receptors [62,65,66]. In plants branched or mixed chains may also play an important role in cell signalling. The master coactivator of plant immunity, NPR1, is regulated by sequential activities of the modular CRL3 ligase (Cullin-RING Ligase 3), the U-box-containing E4 ligase UBE4 (ubiquitin conjugation factor E4) and HECT-type UPL3 and UPL4 ligases (ubiquitin protein ligase 3 and 4) [34–36]. Each of these ligases may generate diverse ubiquitin chain topologies that affect NPR1 activity and function. Initial ubiquitination of NPR1 by CRL3 induces its activity and promotes its association with target promoters, leading to high levels of target gene expression [34,35]. Subsequent chain elongation by UBE4 deactivates NPR1 and promotes recruitment of the proteasome. Curiously, proteasome-bound NPR1 is then further ubiquitinated by the HECT E3 ligases, UPL3 and UPL4, which is necessary for efficient and processive degradation by the proteasome [36]. Even though the ubiquitin chain topologies of NPR1 have not been described yet, the activities of the trio of ubiquitin ligases that modify it, can provide insight into the potential regulatory linkage types. Studies in different eukaryotes have shown that CRL3 targets a broad range of regulatory proteins. While CRL3 has been associated with mono-, K33-linked, and even non-lysine residue ubiquitylation [67], it modifies most of its substrates with K48-linked chains, targeting them for proteasome-mediated degradation [67]. Given that CRL3 activates NPR1, it is likely CRL3 modifies NPR1 with multi-monoubiquitin or K48-linked chains that are too short to trigger recruitment of the proteasome. On the other hand, mammalian UBE4 (UBE4B) promotes or extends both K48- and K63-linked polyubiquitination primed by other E3 enzymes [68]. Since UBE4 triggers the recruitment of the proteasome to the ubiquitinated NPR1, it likely extends CRL3-primed K48-linked chains or generates K63/K48 branched chains that are more efficiently recognized by the proteasome. Finally, unlike CRL3 and UBE4, the ability to build linkage-specific polyubiquitin chains appears to be an intrinsic feature of HECT-type ligases, as they are able to generate distinct ubiquitin chains regardless of the E2 enzymes they pair with [69]. Some examples are NEDD4 family members that synthesize K63 chains [70], while E6AP is a K48-specific enzyme [71], and HUWE1 generates K6-, K11-, and K48- linked polyubiquitin chains [72]. Because of their vital role in promoting proteasome processivity (see review in this issue by Wang and Spoel), HECT-type ligases may generate various mixed or branched linkage topologies on NPR1, dependent on the needs of the proteasome.

## Future perspectives: cross-talk and recognition

The progress made over the last decade in understanding how ubiquitin chains are assembled is astonishing. Much of this knowledge has been gained by identifying novel components, E3 ligases activities, structures of diverse ubiquitin topologies, and ubiquitin readers that decode the ubiquitin code. This knowledge now serves as a solid foundation to understand how the ubiquitin system integrates and modulates responses to environmental signals. Areas that are likely to develop quickly in the coming years include the functional studies of diverse ubiquitin chain topologies, regulation of ubiquitin linkages by other post-tranlational modifications, and how this complex code is recognized by UBD-containing proteins and associated protein complexes.

Complexity of the ubiquitin code is further expanded by post-translational modification of ubiquitin itself by acetylation, phosphorylation, ADP-ribosylation, phosphoribosylation, deamidation, SUMOylation and succinylation, each of them potentially capable of modulating the function of ubiquitin. SUMOylation not only competes with ubiquitin for attachment to substrate lysine residues, direct SUMOylation of ubiquitin chains implies more intricate cross-talk between these two signals [73,74]. Acetylation negatively regulates ubiquitin chain elongation by competing with the acceptor ubiquitin, but may also prevent chain assembly at nearby lysine residues [75]. Phosphorylation of ubiquitin at Ser65 is rare in steady-state conditions but dramatically increases when mitochondria are depolarized [76]. Interestingly, phosphorylation affects the structure of ubiquitin and, consequently, formation of ubiquitin chains by a subset of E2 and E3 enzymes [76,77]. Furthermore, some DUBs are not able to hydrolyse chains that contain Ser65-phospho-ubiquitin [77]. Because many of these post-translational modifications alter charge and surface properties of ubiquitin, they likely have a dramatic impact on functional docking points for UBDs. UBDs interact with their targets in a transient, non-covalent manner and frequently in complex with E3 ligases and DUBs, where UBDs contribute to enzyme functionality and/or substrate selectivity [78]. UBDs utilize diverse surfaces to make contact with the ubiquitin chain. Even though most of the surface of ubiquitin is polar, ubiquitin contains hydrophobic patches essential for its interaction with UBDs, including the most frequently utilized Ile44/Val70 patch and the less common Ile36 and Phe4 patches [79,80]. Even though these represent relatively low-affinity interactions between ubiquitin and UBDs, they are critical for rapid and reversible cellular responses to environmental cues. Moreover, UBDs are highly specific in selecting chain topologies, sensing chain length and can be combined with other UBDs of distinct specificity to generate novel ubiquitin-binding activities [81,82], demonstrating co-evolution between ubiquitin chain topologies and UBDs (Figure 3). Future studies in plants should reveal the functionalities of UBD-containing proteins or the potential post-translational modifications of ubiquitin that may affect ubiquitin-UBD interactions.

From an evolutionary point of view, the ubiquitin code’s complexity is astonishing. Given the increased interest of plant scientist to resolve the complexity of this code, the next decade will be critical in providing frontiers knowledge on our understanding of the ubiquitin system as a signaling hub. Development of methods to monitor *in vivo* dynamic interactions of ubiquitinated proteins, advances in mass spectroscopy, use of ubiquitin chain-specific antibodies, and development of ubiquitin sensors, will provide promising tools to decipher the complex networks of ubiquitin signalling in plants.

## Summary

- Diverse chain topologies constitute a code utilized to modify proteins’ functions in numerous cellular signalling pathways.
- The cooperative action between a large array of E2 conjugating and E3 ligase enzymes allows the formation of homotypic, heterotypic mixed and branched ubiquitin chains.
- The ubiquitin code is determined by the principles of chain assembly: distinct mechanisms of assembly, different chain initiation and elongation events, and optimal positioning of ubiquitin molecules to favour specific linkage types.
- The structures of ubiquitin bound to different ubiquitin-binding domains are revealing the mechanisms underpinning selective and specific signalling by the ubiquitin code.

## Open Access

Open access for this article was enabled by the participation of The University of Edinburgh in an all-inclusive *Read & Publish* agreement with Portland Press and the Biochemical Society under a transformative agreement with JISC.

## Abbreviations

ABA: abscisic acid
BR: brassinosteroids
DUB: deubiquitinase
GA: gibberellic acid
IRF3: interferon regulatory factor 3
JA: jasmonic acid
SA: salicylic acid
UBD: ubiquitin-binding domain
